# Supplementary material for: A Framework for Comparison and Interpretation of Machine Learning Classifiers to Predict Autism on the ABIDE Dataset
Source: Hum Brain Mapp. 2025 Mar 17;46(5):e70190. doi: 10.1002/hbm.70190 (PMC11912182; doi:10.1002/hbm.70190)
Supplement: Supplementary file 1 — Data S1. Supporting Information. [file HBM-46-e70190-s001.pdf]

Supplementary Information: Reproducible comparison and interpretation of machine learning classifiers to predict autism on the ABIDE multimodal dataset

Yilan Dong[1,2], Dafnis Batalle[1,2], Maria Deprez[1]

[1] School of Biomedical Engineering & Imaging Sciences, King's College London, London SE1 7EH, United Kingdom

[2] Department of Forensic and Neurodevelopmental Science, Institute of Psychiatry, Psychology & Neuroscience, King's College London, London SE5 8AF, United Kingdom

Corresponding author:
Yilan Dong
School of Biomedical Engineering & Imaging Sciences, King's College London, London SE1 7EH, United Kingdom
yilan.dong@kcl.ac.uk

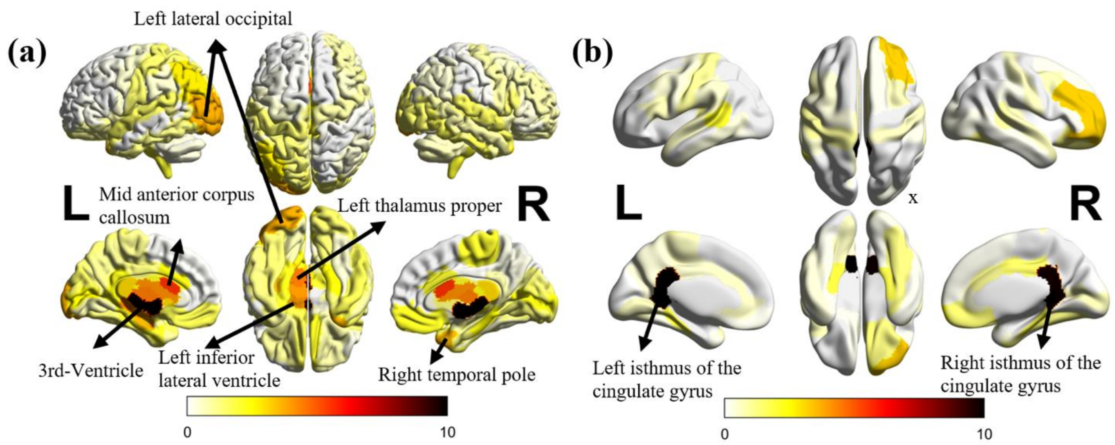

Supplementary Fig. 1 Important structural brain features identified by GCN model contributing to the autism classification task. (a) Cortical + Subcortical areas. (b) White Matter areas.

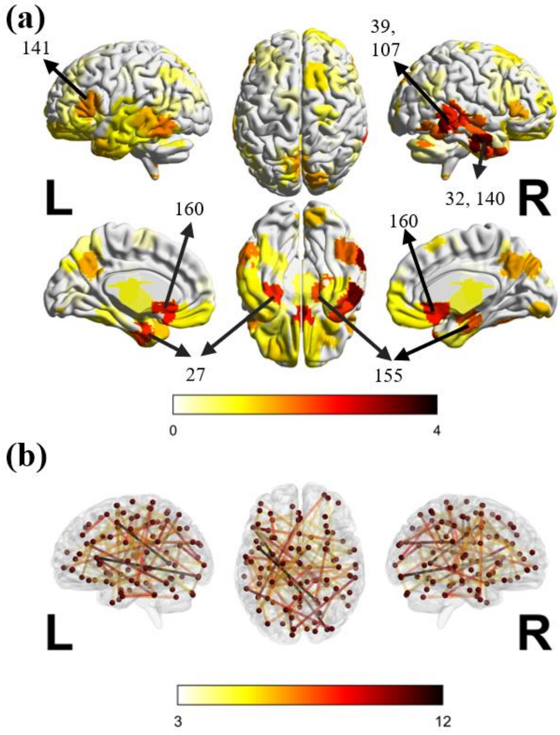

Supplementary Fig. 2 (a) The important brain regions identified from fMRI features by GCN model, with numbers corresponding to regions in the CC200 atlas. (b) Top 100 connections.

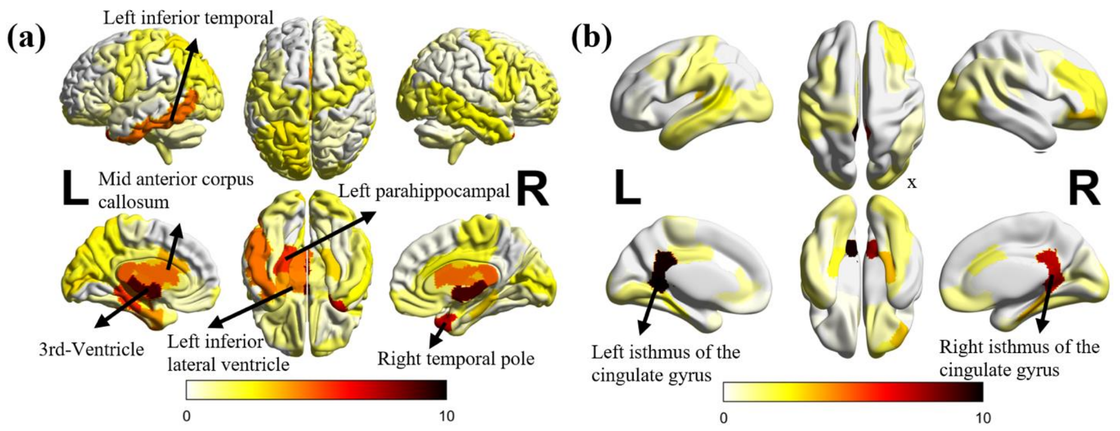

Supplementary Fig. 3 Important structural brain features identified by AE-FCN model contributing to the autism classification task. (a) Cortical + Subcortical areas. (b) White Matter areas.

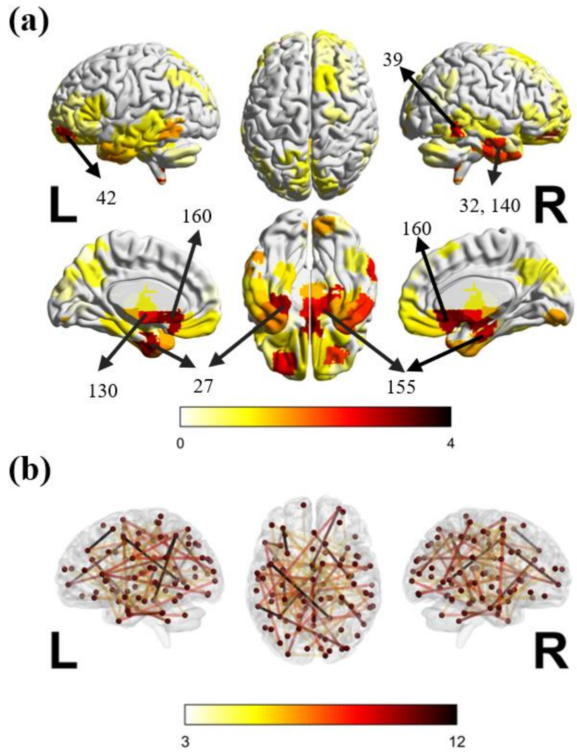

Supplementary Fig. 4 (a) The important brain regions identified from fMRI features by AE-FCN model, with numbers corresponding to regions in the CC200 atlas. (b) Top 100 connections.

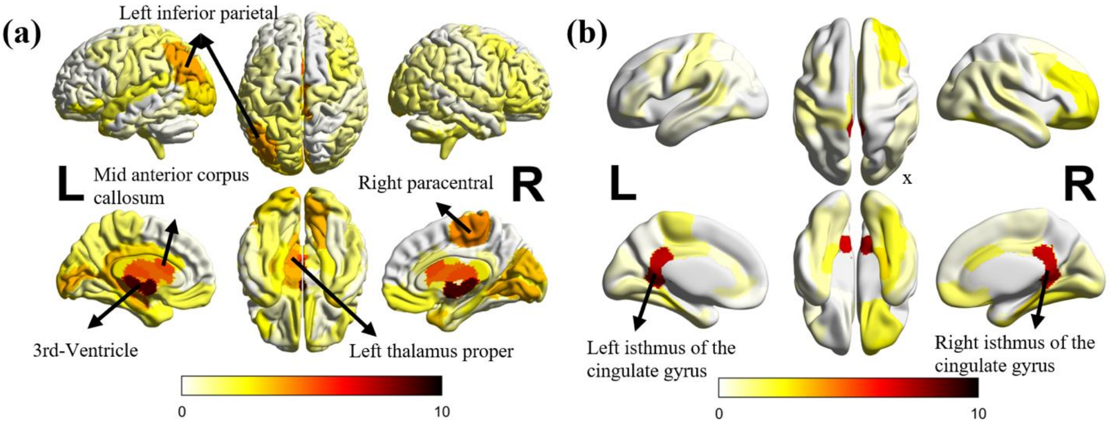

Supplementary Fig. 5 Important structural brain features identified by EV-GCN model contributing to the autism classification task. (a) Cortical + Subcortical areas. (b) White Matter areas.

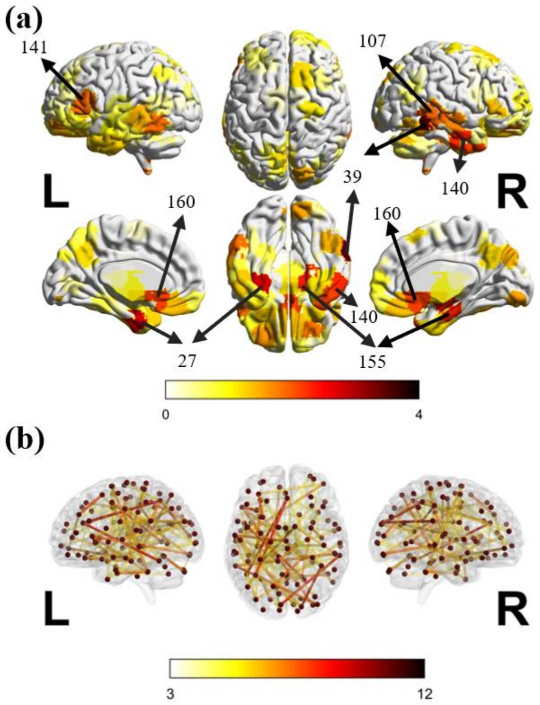

Supplementary Fig. 6 (a) The important brain regions identified from fMRI features by EV-GCN model, with numbers corresponding to regions in the CC200 atlas. (b) Top 100 connections.

Supplementary Table 1. The top 100 most important brain connections

The top 100 connections	Brain regions in CC200 atlas	The corresponding brain regions in AAL atlas
1	101	Temporal_Inf_L: 0.58, Temporal_Mid_L: 0.38
	155	Hippocampus_R: 0.39, ParaHippocampal_R: 0.37, None: 0.11
2	53	Frontal_Inf_Orb_R: 0.88
	127	Frontal_Mid_R: 0.70, Frontal_Sup_R: 0.30
3	72	Temporal_Mid_L: 0.88
	104	Frontal_Sup_Medial_L: 0.70, Frontal_Sup_L: 0.29
4	32	Temporal_Pole_Mid_R: 0.59, Temporal_Inf_R: 0.35
	155	Hippocampus_R: 0.39, ParaHippocampal_R: 0.37, None: 0.11
5	56	Parietal_Inf_L: 0.85
	144	Frontal_Inf_Tri_R: 0.91
6	19	Calcarine_L: 0.37, Cuneus_L: 0.36, Precuneus_L: 0.16
	140	Temporal_Mid_R: 0.65, Temporal_Inf_R: 0.33
7	155	Hippocampus_R: 0.39, ParaHippocampal_R: 0.37, None: 0.11
	198	Fusiform_R: 0.41, ParaHippocampal_R: 0.25, Temporal_Pole_Mid_R: 0.15, Temporal_Inf_R: 0.13
8	17	Frontal_Inf_Oper_R: 0.44, Precentral_R: 0.31, Frontal_Mid_R: 0.13, Frontal_Inf_Tri_R: 0.13
	20	Insula_L: 0.76, Frontal_Inf_Tri_L: 0.12
9	40	Cingulum_Ant_R: 0.44, Cingulum_Ant_L: 0.40
	140	Temporal_Mid_R: 0.65, Temporal_Inf_R: 0.33
10	102	Occipital_Mid_R: 1.00
	160	Olfactory_L: 0.27, Olfactory_R: 0.22
11	26	Occipital_Inf_R: 0.55, Temporal_Inf_R: 0.16, Cerebelum_Crus1_R: 0.11
	102	Occipital_Mid_R: 1.00
12	94	Caudate_R: 0.84, Thalamus_R: 0.14
	124	Frontal_Mid_Orb_R: 0.61, Frontal_Sup_Orb_R: 0.28
13	11	Temporal_Mid_L: 0.96
	32	Temporal_Pole_Mid_R: 0.59, Temporal_Inf_R: 0.35
14	92	Hippocampus_L: 0.41, Amygdala_L: 0.29, ParaHippocampal_L: 0.15
	141	Frontal_Inf_Tri_L: 0.96
15	145	Hippocampus_L: 0.38, Fusiform_L: 0.31, ParaHippocampal_L: 0.16, Temporal_Inf_L: 0.15
	199	ParaHippocampal_L: 0.38, Cerebelum_4_5_L: 0.34, Cerebelum_3_L: 0.18
16	140	Temporal_Mid_R: 0.65, Temporal_Inf_R: 0.33
	155	Hippocampus_R: 0.39, ParaHippocampal_R: 0.37, None: 0.11
17	39	Temporal_Mid_R: 0.67, Temporal_Inf_R: 0.33
	155	Hippocampus_R: 0.39, ParaHippocampal_R: 0.37, None: 0.11
18	47	Caudate_L: 0.45, Putamen_L: 0.39
	135	Caudate_R: 0.85, Putamen_R: 0.11

19	87	Fusiform_R: 0.32, ParaHippocampal_R: 0.23, Temporal_Inf_R: 0.23, Hippocampus_R: 0.22
	96	Postcentral_L: 0.65, Precentral_L: 0.21, Parietal_Sup_L: 0.12
20	70	Calcarine_L: 0.51, Lingual_L: 0.49
	81	Cuneus_R: 0.51, Precuneus_R: 0.31, Occipital_Sup_R: 0.14
21	39	Temporal_Mid_R: 0.67, Temporal_Inf_R: 0.33
	62	Fusiform_R: 0.54, Hippocampus_R: 0.21, ParaHippocampal_R: 0.19
22	100	Temporal_Inf_R: 0.66, Fusiform_R: 0.26
	189	Fusiform_L: 0.51, Cerebelum_6_L: 0.29, Lingual_L: 0.17
23	95	Frontal_Mid_L: 0.72, Frontal_Sup_L: 0.28
	147	Precuneus_L: 0.54, Cuneus_L: 0.33
24	56	Parietal_Inf_L: 0.85
	107	Temporal_Sup_R: 0.50, Temporal_Mid_R: 0.49
25	107	Temporal_Sup_R: 0.50, Temporal_Mid_R: 0.49
	153	Temporal_Sup_R: 0.64, Temporal_Mid_R: 0.33
26	81	Cuneus_R: 0.51, Precuneus_R: 0.31, Occipital_Sup_R: 0.14
	105	Lingual_R: 0.53, Calcarine_R: 0.31
27	99	Temporal_Mid_L: 0.60, Temporal_Inf_L: 0.40
	151	Frontal_Inf_Tri_L: 0.73, Frontal_Mid_L: 0.20
28	26	Occipital_Inf_R: 0.55, Temporal_Inf_R: 0.16, Cerebelum_Crus1_R: 0.11
	175	Occipital_Inf_R: 0.52, Fusiform_R: 0.15, Lingual_R: 0.14, Cerebelum_Crus1_R: 0.14
29	63	Temporal_Inf_L: 0.45, Fusiform_L: 0.36, Cerebelum_Crus1_L: 0.13
	197	Precuneus_L: 0.92
30	48	Hippocampus_R: 0.28, Lingual_R: 0.21, Thalamus_R: 0.20, ParaHippocampal_R: 0.15
	199	ParaHippocampal_L: 0.38, Cerebelum_4_5_L: 0.34, Cerebelum_3_L: 0.18
31	128	SupraMarginal_R: 0.82, Temporal_Sup_R: 0.17
	188	Parietal_Sup_L: 0.61, Precuneus_L: 0.37
32	40	Cingulum_Ant_R: 0.44, Cingulum_Ant_L: 0.40
	72	Temporal_Mid_L: 0.88
33	104	Frontal_Sup_Medial_L: 0.70, Frontal_Sup_L: 0.29
	109	Frontal_Med_Orb_L: 0.37, Frontal_Med_Orb_R: 0.35
34	107	Temporal_Sup_R: 0.50, Temporal_Mid_R: 0.49
	150	Occipital_Mid_L: 0.54, Temporal_Mid_L: 0.46
35	20	Insula_L: 0.76, Frontal_Inf_Tri_L: 0.12
	144	Frontal_Inf_Tri_R: 0.91
36	32	Temporal_Pole_Mid_R: 0.59, Temporal_Inf_R: 0.35
	179	Lingual_L: 0.48, Cerebelum_4_5_L: 0.21, Calcarine_L: 0.14, Precuneus_L: 0.12
37	87	Fusiform_R: 0.32, ParaHippocampal_R: 0.23, Temporal_Inf_R: 0.23, Hippocampus_R: 0.22

	122	Fusiform_L: 0.47, ParaHippocampal_L: 0.23, Hippocampus_L: 0.21
38	140	Temporal_Mid_R: 0.65, Temporal_Inf_R: 0.33
	174	Precuneus_L: 0.46, Precuneus_R: 0.36
39	160	Olfactory_L: 0.27, Olfactory_R: 0.22
	170	Occipital_Mid_R: 0.70, Occipital_Sup_R: 0.30
40	74	Frontal_Inf_Orb_L: 0.90
	92	Hippocampus_L: 0.41, Amygdala_L: 0.29, ParaHippocampal_L: 0.15
41	81	Cuneus_R: 0.51, Precuneus_R: 0.31, Occipital_Sup_R: 0.14
	95	Frontal_Mid_L: 0.72, Frontal_Sup_L: 0.28
42	47	Caudate_L: 0.45, Putamen_L: 0.39
	94	Caudate_R: 0.84, Thalamus_R: 0.14
43	42	Frontal_Mid_Orb_L: 0.78, Frontal_Inf_Orb_L: 0.14
	78	Temporal_Pole_Mid_L: 0.54, Temporal_Pole_Sup_L: 0.40
44	141	Frontal_Inf_Tri_L: 0.96
	155	Hippocampus_R: 0.39, ParaHippocampal_R: 0.37, None: 0.11
45	39	Temporal_Mid_R: 0.67, Temporal_Inf_R: 0.33
	86	Cerebelum_9_R: 0.48, None: 0.29, Vermis_10: 0.14
46	59	Insula_R: 0.61, Putamen_R: 0.20, Frontal_Inf_Tri_R: 0.11
	144	Frontal_Inf_Tri_R: 0.91
47	75	Frontal_Mid_R: 0.58, Frontal_Sup_R: 0.42
	196	Temporal_Pole_Sup_L: 0.62, Frontal_Inf_Orb_L: 0.22, Insula_L: 0.11
48	63	Temporal_Inf_L: 0.45, Fusiform_L: 0.36, Cerebelum_Crus1_L: 0.13
	136	Parietal_Sup_L: 0.50, Precuneus_L: 0.50
49	50	Precentral_L: 0.64, Frontal_Sup_L: 0.27
	150	Occipital_Mid_L: 0.54, Temporal_Mid_L: 0.46
50	141	Frontal_Inf_Tri_L: 0.96
	145	Hippocampus_L: 0.38, Fusiform_L: 0.31, ParaHippocampal_L: 0.16, Temporal_Inf_L: 0.15
51	49	Temporal_Mid_R: 0.64, Temporal_Inf_R: 0.34
	62	Fusiform_R: 0.54, Hippocampus_R: 0.21, ParaHippocampal_R: 0.19
52	81	Cuneus_R: 0.51, Precuneus_R: 0.31, Occipital_Sup_R: 0.14
	89	Calcarine_R: 0.43, Lingual_R: 0.35
53	100	Temporal_Inf_R: 0.66, Fusiform_R: 0.26
	102	Occipital_Mid_R: 1.00
54	26	Occipital_Inf_R: 0.55, Temporal_Inf_R: 0.16, Cerebelum_Crus1_R: 0.11
	160	Olfactory_L: 0.27, Olfactory_R: 0.22
55	4	Temporal_Sup_L: 0.33, Putamen_L: 0.28, Insula_L: 0.25
	198	Fusiform_R: 0.41, ParaHippocampal_R: 0.25, Temporal_Pole_Mid_R: 0.15, Temporal_Inf_R: 0.13
56	6	Cingulum_Mid_R: 0.60, Precuneus_R: 0.21
	147	Precuneus_L: 0.54, Cuneus_L: 0.33
57	63	Temporal_Inf_L: 0.45, Fusiform_L: 0.36, Cerebelum_Crus1_L: 0.13

	168	Frontal_Mid_R: 0.96
58	58	Precuneus_L: 0.31, Precuneus_R: 0.28, Vermis_4_5: 0.14
	109	Frontal_Med_Orb_L: 0.37, Frontal_Med_Orb_R: 0.35
59	76	Cingulum_Mid_L: 0.86
	107	Temporal_Sup_R: 0.50, Temporal_Mid_R: 0.49
60	119	Rolandic_Oper_R: 0.42, Frontal_Inf_Oper_R: 0.23, Temporal_Sup_R: 0.19
	188	Parietal_Sup_L: 0.61, Precuneus_L: 0.37
61	14	Angular_R: 0.46, Temporal_Mid_R: 0.36
	81	Cuneus_R: 0.51, Precuneus_R: 0.31, Occipital_Sup_R: 0.14
62	11	Temporal_Mid_L: 0.96
	78	Temporal_Pole_Mid_L: 0.54, Temporal_Pole_Sup_L: 0.40
63	65	Postcentral_R: 0.33, Paracentral_Lobule_R: 0.26, Precuneus_R: 0.22, Parietal_Sup_R: 0.19
	96	Postcentral_L: 0.65, Precentral_L: 0.21, Parietal_Sup_L: 0.12
64	57	Frontal_Inf_Orb_L: 0.72, Frontal_Mid_Orb_L: 0.25
	127	Frontal_Mid_R: 0.70, Frontal_Sup_R: 0.30
65	25	Frontal_Mid_R: 0.69, Frontal_Inf_Tri_R: 0.31
	183	Frontal_Mid_L: 0.43, Frontal_Sup_L: 0.42
66	39	Temporal_Mid_R: 0.67, Temporal_Inf_R: 0.33
	58	Precuneus_L: 0.31, Precuneus_R: 0.28, Vermis_4_5: 0.14
67	71	Frontal_Inf_Orb_R: 0.55, Insula_R: 0.30
	112	Insula_L: 0.34, Frontal_Inf_Orb_L: 0.29, Temporal_Pole_Sup_L: 0.26
68	153	Temporal_Sup_R: 0.64, Temporal_Mid_R: 0.33
	194	None: 0.71, Vermis_3: 0.14
69	38	Frontal_Mid_R: 0.77, Frontal_Inf_Tri_R: 0.14
	113	Frontal_Mid_Orb_R: 0.42, Frontal_Mid_R: 0.28, Frontal_Inf_Orb_R: 0.25
70	160	Olfactory_L: 0.27, Olfactory_R: 0.22
	175	Occipital_Inf_R: 0.52, Fusiform_R: 0.15, Lingual_R: 0.14, Cerebelum_Crus1_R: 0.14
71	32	Temporal_Pole_Mid_R: 0.59, Temporal_Inf_R: 0.35
	97	Occipital_Mid_L: 0.87, Occipital_Sup_L: 0.11
72	144	Frontal_Inf_Tri_R: 0.91
	153	Temporal_Sup_R: 0.64, Temporal_Mid_R: 0.33
73	27	Fusiform_L: 0.45, Temporal_Inf_L: 0.21, ParaHippocampal_L: 0.13
	57	Frontal_Inf_Orb_L: 0.72, Frontal_Mid_Orb_L: 0.25
74	19	Calcarine_L: 0.37, Cuneus_L: 0.36, Precuneus_L: 0.16
	147	Precuneus_L: 0.54, Cuneus_L: 0.33
75	51	Frontal_Med_Orb_L: 0.27, Rectus_L: 0.23, Frontal_Med_Orb_R: 0.21, Rectus_R: 0.18
	103	Cerebelum_6_L: 0.46, Cerebelum_4_5_L: 0.32
76	10	Cerebelum_Crus1_R: 0.77, Cerebelum_6_R: 0.11

	117	Temporal_Mid_L: 0.82, Temporal_Sup_L: 0.18
77	23	Frontal_Inf_Tri_L: 0.57, Frontal_Mid_L: 0.42
	42	Frontal_Mid_Orb_L: 0.78, Frontal_Inf_Orb_L: 0.14
78	39	Temporal_Mid_R: 0.67, Temporal_Inf_R: 0.33
	99	Temporal_Mid_L: 0.60, Temporal_Inf_L: 0.40
79	23	Frontal_Inf_Tri_L: 0.57, Frontal_Mid_L: 0.42
	91	Frontal_Sup_Medial_L: 0.56, Frontal_Sup_Medial_R: 0.34
80	39	Temporal_Mid_R: 0.67, Temporal_Inf_R: 0.33
	97	Occipital_Mid_L: 0.87, Occipital_Sup_L: 0.11
81	70	Calcarine_L: 0.51, Lingual_L: 0.49
	164	Frontal_Inf_Tri_R: 0.62, Frontal_Inf_Oper_R: 0.36
82	42	Frontal_Mid_Orb_L: 0.78, Frontal_Inf_Orb_L: 0.14
	43	Temporal_Inf_L: 0.50, Temporal_Pole_Mid_L: 0.26, None: 0.16
83	18	Thalamus_R: 0.99
	107	Temporal_Sup_R: 0.50, Temporal_Mid_R: 0.49
84	20	Insula_L: 0.76, Frontal_Inf_Tri_L: 0.12
	39	Temporal_Mid_R: 0.67, Temporal_Inf_R: 0.33
85	155	Hippocampus_R: 0.39, ParaHippocampal_R: 0.37, None: 0.11
	193	Frontal_Sup_R: 0.50, Frontal_Sup_Medial_R: 0.42
86	133	Frontal_Sup_Medial_L: 0.62, Frontal_Sup_L: 0.36
	155	Hippocampus_R: 0.39, ParaHippocampal_R: 0.37, None: 0.11
87	54	Cuneus_L: 0.62, Occipital_Sup_L: 0.20
	129	Temporal_Sup_L: 0.34, Temporal_Mid_L: 0.26, Temporal_Pole_Sup_L: 0.17
88	87	Fusiform_R: 0.32, ParaHippocampal_R: 0.23, Temporal_Inf_R: 0.23, Hippocampus_R: 0.22
	179	Lingual_L: 0.48, Cerebelum_4_5_L: 0.21, Calcarine_L: 0.14, Precuneus_L: 0.12
89	129	Temporal_Sup_L: 0.34, Temporal_Mid_L: 0.26, Temporal_Pole_Sup_L: 0.17
	140	Temporal_Mid_R: 0.65, Temporal_Inf_R: 0.33
90	118	Cerebelum_6_R: 0.70, Cerebelum_4_5_R: 0.18
	166	Angular_R: 0.85, Occipital_Mid_R: 0.13
91	75	Frontal_Mid_R: 0.58, Frontal_Sup_R: 0.42
	106	Frontal_Mid_R: 0.72, Frontal_Sup_R: 0.28
92	20	Insula_L: 0.76, Frontal_Inf_Tri_L: 0.12
	115	Precentral_R: 0.56, Frontal_Mid_R: 0.44
93	6	Cingulum_Mid_R: 0.60, Precuneus_R: 0.21
	81	Cuneus_R: 0.51, Precuneus_R: 0.31, Occipital_Sup_R: 0.14
94	11	Temporal_Mid_L: 0.96
	51	Frontal_Med_Orb_L: 0.27, Rectus_L: 0.23, Frontal_Med_Orb_R: 0.21, Rectus_R: 0.18
95	27	Fusiform_L: 0.45, Temporal_Inf_L: 0.21, ParaHippocampal_L: 0.13

	114	Occipital_Mid_L: 0.32, Occipital_Sup_L: 0.27, Parietal_Sup_L: 0.26, Parietal_Inf_L: 0.15
96	114	Occipital_Mid_L: 0.32, Occipital_Sup_L: 0.27, Parietal_Sup_L: 0.26, Parietal_Inf_L: 0.15
	160	Olfactory_L: 0.27, Olfactory_R: 0.22
97	32	Temporal_Pole_Mid_R: 0.59, Temporal_Inf_R: 0.35
	178	Putamen_R: 0.69, Pallidum_R: 0.21
98	140	Temporal_Mid_R: 0.65, Temporal_Inf_R: 0.33
	178	Putamen_R: 0.69, Pallidum_R: 0.21
99	57	Frontal_Inf_Orb_L: 0.72, Frontal_Mid_Orb_L: 0.25
	122	Fusiform_L: 0.47, ParaHippocampal_L: 0.23, Hippocampus_L: 0.21
100	32	Temporal_Pole_Mid_R: 0.59, Temporal_Inf_R: 0.35
	106	Frontal_Mid_R: 0.72, Frontal_Sup_R: 0.28

Supplementary Table 2. The top 100 most important structural volumetric features

The top 100 structural features	Desikan-Killiany atlas regions	feature names
1	3rd-Ventricle	Volume
2	wm-lh-isthmuscingulate	Intensity normMin
3	Left-Inf-Lat-Vent	Volume
4	3rd-Ventricle	Number of Voxels
5	wm-rh-isthmuscingulate	Intensity normRange
6	ctx-rh-temporalpole	CurvInd
7	wm-rh-isthmuscingulate	Intensity normMin
8	CC_Mid_Anterior	Intensity normMin
9	wm-lh-isthmuscingulate	Intensity normRange
10	wm-lh-isthmuscingulate	Itensity normStdDev
11	Left-VentralDC	Volume
12	Left-Thalamus	Intensity normMax
13	Left-Inf-Lat-Vent	Number of Voxels
14	4th-Ventricle	Itensity normStdDev
15	wm-rh-parahippocampal	Volume
16	CSF	Volume
17	ctx-rh-paracentral	ThickAvg
18	Left-VentralDC	Number of Voxels
19	Left-choroid-plexus	Intensity normMean
20	non-WM-hypointensities	Itensity normStdDev
21	Left-Lateral-Ventricle	Itensity normStdDev
22	ctx-lh-pericalcarine	MeanCurv
23	CC_Mid_Anterior	Intensity normRange
24	ctx-rh-parahippocampal	NumVert
25	Left-Caudate	Intensity normMax
26	CSF	Number of Voxels
27	CC_Posterior	Intensity normMin
28	ctx-rh-pericalcarine	MeanCurv
29	Left-Pallidum	Intensity normMax
30	ctx-lh-isthmuscingulate	MeanCurv
31	ctx-lh-cuneus	GausCurv
32	wm-rh-parahippocampal	Number of Voxels
33	Left-Hippocampus	Itensity normStdDev
34	ctx-lh-inferiorparietal	FoldInd
35	ctx-lh-entorhinal	FoldInd
36	ctx-lh-pericalcarine	GausCurv
37	ctx-rh-parahippocampal	GrayVol
38	CC_Central	Volume
39	ctx-lh-pericalcarine	CurvInd
40	ctx-rh-pericalcarine	ThickStd
41	wm-rh-rostralmiddlefrontal	Intensity normMin

42	ctx-lh-superiortemporal	ThickAvg
43	ctx-lh-caudalanteriorcingulate	FoldInd
44	ctx-lh-superiorparietal	CurvInd
45	ctx-lh-parahippocampal	NumVert
46	ctx-rh-temporalpole	FoldInd
47	ctx-lh-entorhinal	ThickStd
48	ctx-lh-temporalpole	ThickStd
49	ctx-lh-lateraloccipital	ThickAvg
50	Left-Lateral-Ventricle	Volume
51	ctx-lh-parahippocampal	CurvInd
52	CC_Mid_Posterior	Intensity normMin
53	wm-lh-isthmuscingulate	Number of Voxels
54	wm-lh-parahippocampal	Volume
55	Left-Lateral-Ventricle	Number of Voxels
56	ctx-lh-entorhinal	CurvInd
57	ctx-rh-caudalanteriorcingulate	GausCurv
58	WM-hypointensities	Intensity normMean
59	Right-Inf-Lat-Vent	Itensity normStdDev
60	ctx-lh-posteriorcingulate	MeanCurv
61	ctx-lh-parahippocampal	SurfArea
62	wm-lh-bankssts	Volume
63	Left-Hippocampus	Intensity normMax
64	wm-lh-bankssts	Itensity normStdDev
65	Right-UnsegmentedWhiteMatter	Itensity normStdDev
66	ctx-rh-parahippocampal	SurfArea
67	wm-rh-rostralmiddlefrontal	Intensity normRange
68	CC_Central	Number of Voxels
69	ctx-rh-parsopercularis	MeanCurv
70	ctx-lh-precuneus	MeanCurv
71	CC_Mid_Anterior	Itensity normStdDev
72	ctx-rh-medialorbitofrontal	ThickStd
73	ctx-lh-precuneus	GausCurv
74	wm-rh-lateraloccipital	Intensity normMax
75	ctx-lh-parahippocampal	ThickStd
76	wm-lh-parahippocampal	Number of Voxels
77	Right-Lateral-Ventricle	Itensity normStdDev
78	ctx-rh-medialorbitofrontal	MeanCurv
79	Left-Amygdala	Volume
80	ctx-lh-parahippocampal	GrayVol
81	Right-UnsegmentedWhiteMatter	Intensity normMean
82	wm-rh-entorhinal	Itensity normStdDev
83	ctx-rh-pericalcarine	FoldInd
84	wm-lh-isthmuscingulate	Volume

85	wm-lh-transversetemporal	Number of Voxels
86	Left-Hippocampus	Intensity normRange
87	ctx-lh-paracentral	NumVert
88	ctx-lh-precuneus	CurvInd
89	Brain-Stem	Itensity normStdDev
90	ctx-rh-entorhinal	ThickStd
91	ctx-rh-inferiortemporal	CurvInd
92	ctx-rh-lateraloccipital	ThickAvg
93	ctx-rh-cuneus	GrayVol
94	ctx-rh-frontalpole	ThickAvg
95	ctx-rh-superiortemporal	GrayVol
96	ctx-lh-inferiorparietal	CurvInd
97	ctx-lh-transversetemporal	GrayVol
98	wm-lh-middletemporal	Intensity normMax
99	wm-lh-bankssts	Number of Voxels
100	ctx-lh-bankssts	MeanCurv

Supplementary Table 3. Experiment results of the SVM model with a 'linear' kernel using six different feature sets

SVM 'linear'	No ensemble			Max voting	
	Validation	Test	AUC	Test	AUC
sMRI	62.64%	59.77%	0.618	62.53%	0.66
sMRI+non-imaging	64.02%	61.15%	0.638	63.91%	0.679
fMRI	68.05%	66.32%	0.711	68.74%	0.74
fMRI+non-imaging	68.05%	66.09%	0.712	68.97%	0.742
sMRI+fMRI	69.66%	66.55%	0.724	68.62%	0.756
sMRI+fMRI+non-imaging	69.54%	66.67%	0.725	69.31%	0.757
